# Allosteric inhibition enhances the efficacy of ABL kinase inhibitors to target unmutated BCR-ABL and BCR-ABL-T315I

**DOI:** 10.1186/1471-2407-12-411

**Published:** 2012-09-17

**Authors:** Afsar Ali Mian, Anna Metodieva, Susanne Badura, Mamduh Khateb, Nili Ruimi, Yousef Najajreh, Oliver Gerhard Ottmann, Jamal Mahajna, Martin Ruthardt

**Affiliations:** 1 Department of Hematology, Goethe University, Frankfurt, Germany; 2 Cancer Drug Discovery, Migal-Galilee Technology Center, Kiryat Shmona, Israel; 3 Faculty of Pharmacy, Al-Quds University, Jerusalem-Abu Dies, Palestine; 4 Departments of Nutritional Sciences, Tel Hai Academic College, Kiryat Shmona, Israel; 5 Labor für Tumorstammzellbiologie, Med. Klinik II/Hämatologie, Klinikum der Goethe Universität Frankfurt, Theodor Stern Kai 7, 60590, Frankfurt, Germany

**Keywords:** Philadelphia chromosome, BCR/ABL, “gatekeeper” mutation T315I, Allosteric inhibition, Abl kinase inhibitors, Molecular therapy

## Abstract

**Background:**

Chronic myelogenous leukemia (CML) and Philadelphia chromosome-positive (Ph+) acute lymphatic leukemia (Ph + ALL) are caused by the t(9;22), which fuses *BCR* to *ABL* resulting in deregulated ABL-tyrosine kinase activity. The constitutively activated BCR/ABL-kinase “escapes” the auto-inhibition mechanisms of c-ABL, such as allosteric inhibition. The ABL-kinase inhibitors (AKIs) Imatinib, Nilotinib or Dasatinib, which target the ATP-binding site, are effective in Ph + leukemia. Another molecular therapy approach targeting BCR/ABL restores allosteric inhibition. Given the fact that all AKIs fail to inhibit BCR/ABL harboring the ‘gatekeeper’ mutation T315I, we investigated the effects of AKIs in combination with the allosteric inhibitor GNF2 in Ph + leukemia.

**Methods:**

The efficacy of this approach on the leukemogenic potential of BCR/ABL was studied in Ba/F3 cells, primary murine bone marrow cells, and untransformed Rat-1 fibroblasts expressing BCR/ABL or BCR/ABL-T315I as well as in patient-derived long-term cultures (PDLTC) from Ph + ALL-patients.

**Results:**

Here, we show that GNF-2 increased the effects of AKIs on unmutated BCR/ABL. Interestingly, the combination of Dasatinib and GNF-2 overcame resistance of BCR/ABL-T315I in all models used in a synergistic manner.

**Conclusions:**

Our observations establish a new approach for the molecular targeting of BCR/ABL and its resistant mutants using a combination of AKIs and allosteric inhibitors.

## Background

Chronic myeloid leukemia (CML) and 30% of adult acute lymphatic leukemia (ALL) are characterized by the Philadelphia chromosome (Ph+), which is the cytogenetic correlate of the (9;22) chromosomal translocation. The t(9;22) leads to the fusion of the *breakpoint cluster region* (*BCR*) gene and the *Abelson tyrosine kinase* (ABL1). BCR/ABL results in a deregulated and constitutively activated tyrosine kinase, which is responsible for the induction of the phenotype of Ph + leukemia. BCR/ABL constitutively activates several signaling pathways leading to uncontrolled proliferation and inhibition of apoptosis. The expression of BCR/ABL is sufficient for the initiation and maintenance of early stage CML and the “CML-like disease” in mice [1,2].

Selective targeting of BCR/ABL by ABL-kinase inhibitors (AKI) such as Imatinib, Nilotinib or Dasatinib, all competitive ATP-analogues, leads to durable cytogenetic and molecular remissions in the majority of CML patients in the early chronic phase of the disease. However, unsatisfactory responses in advanced disease stages, resistance and long-term tolerability of BCR/ABL inhibitors represent major clinical problems. In fact, advanced CML and Ph + ALL respond only transiently to AKIs [3,4]. Secondary resistance is mostly caused by the acquisition of point mutations in BCR/ABL that interfere with the affinity for these ATP competitors. The second-generation inhibitors Nilotinib and Dasatinib target most resistant BCR/ABL mutants [5,6] with the exception of the “gatekeeper” mutation T315I. T315I is the most clinically relevant mutation because it confers a global resistance against all available molecular therapy approaches [3,4].

The activation status of wild-type c-ABL is finely regulated by several regulation signals. Myristoylation of the N-terminus of c-ABL is involved in the regulation of the ABL kinase activity. The N-terminus of ABL is myristoylated, and the myristate residue binds to a hydrophobic pocket in the kinase domain - the myristoyl-binding pocket (MBP) – in a process called “capping”. The “capping” leads to conformational changes that allow the intramolecularly docking of the “SRC homology 2 domain” to the kinase domain. Hence, c-ABL adopts an auto-inhibited conformation. The absence of an N-terminal myristoylated domain activates c-ABL consistent with its auto-regulatory role. In the context of the t(9;22), the N-terminal auto-inhibitory “Cap” region is substituted by the BCR portion of the fusion protein. The absence of the “Cap” region allows the BCR/ABL to “escape” auto-inhibition contributing to the constitutive activation of its kinase activity [7].

We have recently shown that the allosteric inhibition increases the sensitivity of BCR/ABL-T315I towards the inhibition of oligomerization most likely by interfering with the overall confirmation of the kinase [4]. Given the fact that the resistance against AKIs in the BCR/ABL-T315I mutant is a problem of the accessibility of the ATP-binding site in the kinase domain, we analyzed the influence of the allosteric inhibition on the response of BCR/ABL-T315I towards AKIs. Preliminary data showed the best effect for Dasatinib compared to Nilotinib or Imatinib. Therefore, we analyzed whether it was possible to enhance the response and to overcome the resistance of the BCR/ABL-T315I mutant by combining the allosteric inhibition of GNF-2 with Dasatinib.

## Methods

### Plasmids

The cDNAs encoding BCR/ABL and BCR/ABL-T315I have been previously described (3). All retroviral expression vectors used in this study were based on the bi-cistronic PINCO vector.

### Cell lines and patient-derived long-term cultures

The Ba/F3 and Rat-1 cells were obtained from the German Collection of Microorganisms and Cell Cultures (DSMZ, Braunschweig, Germany) and were maintained as previously described (3). Ph + ALL patient derived long term cultures (PDLTCs) expressing BCR-ABL-T315I (KÖ) were obtained from a patient enrolled in the German Multi-Center Study Group for acute lymphatic leukemia of the adult (GMALL 07/2003) upon informed and written consent [8] and were maintained in a serum–free medium consisting of IMDM supplemented with 1 mg/mL of bovine insulin, 5x10^-5^ M β–mercaptoethanol (Sigma, Steinheim, Germany), 200 mg/mL Fe –saturated human apo–transferrin (Invitrogen, Karlsruhe, Germany), 0.6% human serum albumin (Sanquin, Amsterdam, The Netherlands), 2.0 mM L–glutamine and 20 mg/mL cholesterol (Sigma) [9]. Proliferation was assessed with the XTT proliferation kit (Roche, Mannheim, Germany) according to the manufacturer’s instructions.

### Isolation of Sca1^+^/lin^-^ hematopoietic stem and progenitor cells (HSPCs)

Sca1^+^/lin^-^ HSPCs were isolated from 8- to 12-week-old female C57BL/6 N mice (Janvier, St. Berthevin, France) after euthanization by CO_2_ asphyxiation. Bone marrow (BM) was harvested from the femur and tibia by flushing the bones with a syringe and a 26-gauge needle. Sca1^+^ cells were purified by immunomagnetic beads using MACS cell separation columns according to the manufacturer’s instructions (Miltenyi, Bergisch-Gladbach, Germany). Prior to subsequent use, the purified cells were pre-stimulated for 2 days in DMEM supplemented with 10% FCS (Hyclone/Perbio Science, Bonn Germany), 1% L-Glutamine, 1% Penicillin/Streptomycin, mIL-3 (20 ng/mL), mIL-6 (20 ng/mL) and mSCF (100 ng/mL) (Cell Concepts, Umkirch, Germany).

### Transfection and retroviral infection

Ecotropic retroviral supernatants were obtained after transfection of Phoenix packaging cells as described earlier [3]. For infection of target cells, Retronectin® (Takara Bio Inc., Otsu, Japan) was used to enhance infection efficiency according to the manufacturer’s instructions. Then, 2x10^5^ target cells were seeded per well. Infection efficiency was measured after 48 h by determining the percentage of GFP positive cells using flow cytometry.

### Transformation assays

Soft agar and focus formation assays were performed using untransformed Rat-1 fibroblasts retro virally transduced with PINCO vectors harboring unmutated BCR/ABL or BCR/ABL-T315I. Six-well plates were filled with DMEM supplemented with 10% FCS and 0.5% bacto-agar (DIFCO Laboratories, Detroit, MI, USA) (2 ml per well). Then, 5x10^3^ transduced Rat-1 cells were suspended in “top-agar” (DMEM supplemented with 10% FCS and 0.25% bacto-agar) (1 ml per well) and stacked in the wells. Colonies were counted after 15 days of incubation at 37°C and 5% CO_2_. For focus formation assays, 4x10^4^ transduced Rat-1 cells were plated per well of a 24-well plate. Foci were stained after 15 days using 1% crystal violet (Sigma).

### Colony assays on HSPCs

At day 5 post-infection, Sca1^+^ cells were plated at 5x10^3^ cells/mL in methyl-cellulose either with mIL-3 (20 ng/mL), mIL-6 (20 ng/mL) and mSCF (100 ng/mL) or without cytokines (Stem Cells Inc., Cambridge, UK). The number of colony forming units (CFUs) was determined 10 days after plating and normalized according to the transduction efficiency.

### Western blotting

Western blot analysis was performed according to widely accepted protocols. The following antibodies were used: anti-ABL (α-ABL) (St. Cruz Biotechnology, Santa Cruz, CA, USA), anti-phosphorylated ABL specific for the phosphorylated tyrosine residue 245 (α-p-ABL-Y245) (Cell Signaling, Boston, MA, USA), anti-BCR (α-BCR) (St. Cruz Biotechnology), anti-phosphorylated BCR specific for the phosphorylated tyrosine residue 177 (α-p-BCR-Y177), anti-Crkl, and anti-phosphorylated Crkl (Cell Signaling).

### Statistical analysis

Differences in response rates towards different concentrations of a single inhibitor or inhibitors in combination were analyzed by Student′s t-tests. Statistical analyses were performed using the GraphPad Prism (GraphPad Software, San Diego, CA) software package. Evaluation of the character of the combined effects was performed according to the three dimensional model of Prichard and Shipman using MacSynergy software [10].

## Results

### The allosteric inhibitor GNF-2 improves the response of unmutated BCR/ABL to AKIs

**Figure 1:**
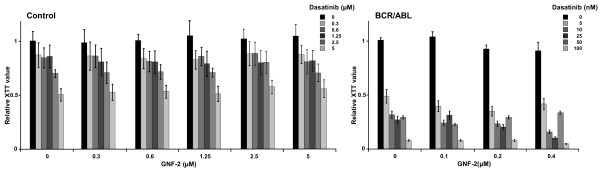
**Effects of the allosteric inhibitor (GNF-2) and Abl kinase inhibitor (Dasatinib) on Ba/F3 cells expressing unmutated BCR/ABL. **(**A**) XTT assay using Ba/F3 cells expressing empty vector upon exposure to 0.3, 0.6, 1.25, 2.5 and 5 μM GNF-2 and 0.3, 0.6, 1.25, 2.5 and 5 μM Dasatinib. (**B**) XTT assay using Ba/F3 cells expressing BCR/ABL upon exposure to 0.1, 0.2 and 0.4 μM GNF-2 and 5, 10, 25, 50 and 100 nM Dasatinib. Proliferation status was determined by the metabolic activity of cells given by the reduction rate of XTT to formazan. The means +/- SD of triplicates from one representative experiment out of three performed are given.

Unmutated BCR/ABL can be efficiently inhibited not only by AKIs but also by allosteric inhibitors such as GNF-2 or GNF-5 [4,11,12]. To determine whether the allosteric inhibition can improve the response of BCR/ABL-positive cells to AKIs, we exposed Ba/F3 cells previously rendered factor-independent by the expression of BCR/ABL to Dasatinib and GNF-2 at concentrations of 5 to 100 nM and 0.1 to 0.4 μM, respectively, upon factor withdrawal. Proliferation/cytotoxicity was assessed by an XTT assay. Ba/F3 cells transduced with empty vector in the presence of mIL-3 were used as a control. As shown in Figure 1, GNF-2 and Dasatinib only affected growth of control cells at the very highest concentrations excluding an unspecific cytotoxic effect of the compounds and their combination (Figure 1). The combination with GNF-2 accelerated and intensified the effects of Dasatinib on the BA/F3-BCR/ABL cells, suggesting a combinatorial effect upon factor withdrawal in these cells (Figure 1).

Taken together, these data suggest that the inhibition effect of Dasatinib is enhanced by GNF-2 in cells expressing unmutated BCR/ABL.

### The combination of GNF-2 with dasatinib efficiently abolishes the BCR/ABL-T315I-mediated factor-independent growth of Ba/F3 cells

**Figure 2:**
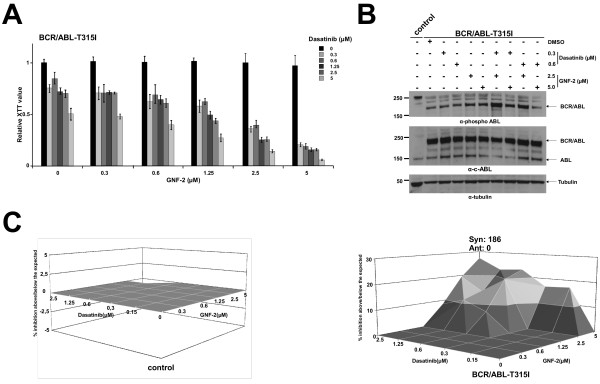
**Effects of the allosteric inhibitor (GNF-2) and Abl kinase inhibitor (Dasatinib) on Ba/F3 cells expressing “gatekeeper” mutation T315I. **(**A**) XTT assay using Ba/F3 cells expressing BCR/ABL-T315I upon exposure to 0.3, 0.6, 1.25, 2.5 and 5 μM GNF-2 and 0.3, 0.6, 1.25, 2.5 and 5 μM Dasatinib. Proliferation status was determined by the metabolic activity of cells given by the reduction rate of XTT to formazan. The means +/- SD of triplicates from one representative experiment out of three performed are given. (**B**) Western blot analysis of Ba/F3 cells expressing the indicated transgenes using antibodies directed against the indicated proteins. (**C**) Calculation of the combined effect by the MacSynergy program.

The major clinical challenge in Ph + leukemia is the drug resistance due to the “gatekeeper” mutation T315I. T315I confers a nearly global resistance to all molecular therapy approaches that target BCR/ABL. Neither GNF-2 nor AKIs have any effect on cells transformed by BCR/ABL-T315I. To analyze whether the combination of allosteric inhibition with AKIs is able to inhibit BCR/ABL-T315I, we exposed Ba/F3 cells expressing BCR/ABL-T315I to increasing concentrations of Dasatinib (0.3, 0.6, 1.25, 2.5 and 5 μM) and GNF-2 (0.3, 0.6, 1.25, 2.5 and 5 μM). Cytotoxicity and proliferation were assessed by the XTT assay. Here, we show that only the combination of GNF-2 and Dasatinib inhibited BCR/ABL-T315I-dependent cell growth with a very high synergy index of 186 (calculated combination index)(Figure 2A-C), whereas Dasatinib alone inhibited growth only at the very highest concentrations. For example, at a GNF-2 concentration of 2 μM, Dasatinib inhibits BCR/ABL-T315I-dependent proliferation with an IC50 of 300 nM without affecting Ba/F3 control cells (Figure 2C). This effect is due to the capacity of the two compounds to efficiently reduce the autophosphorylation of BCR/ABL (Figure 2A).

Taken together, these data suggest that the allosteric inhibition sensitizes BCR/ABL cells harboring the “gatekeeper” mutation T315I towards the ATP analogue Dasatinib.

### The combination of GNF-2 and dasatinib inhibited the growth of Ph + lymphatic PDLTCs expressing BCR/ABL-T315I

**Figure 3:**
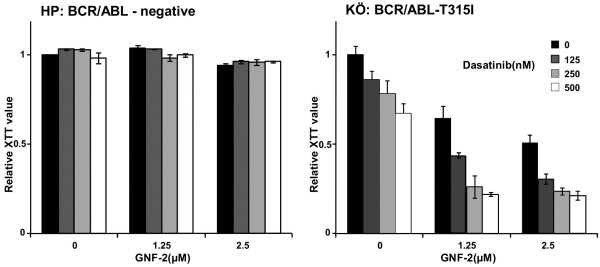
**Effects of GNF-2 and Dasatinib on PDLTCs of Ph + ALL patients expressing BCR/AB-T315I.** Proliferation/citotoxicity was determined by the metabolic activity of cells given by the reduction rate of XTT to formazan. The means +/- SD of triplicates from one representative experiment out of three performed are shown. (**A**) XTT assay using Ph- PDLTCs (HP) upon exposure to 1.25, 2.5 and 5 μM GNF-2 and 125, 25 and 500 nM Dasatinib. (**B**) XTT assay using Ph + PDLTCs (KÖ) expressing BCR/ABL-T315I upon exposure to 1.25, 2.5 and 5 μM GNF-2 and 125, 250 and 500 nM Dasatinib.

Ph + ALL expressing BCR/ABL-T315I is not fully represented in cell lines. Therefore, we tested the response of PDLTCs from Ph + ALL patients expressing BCR/ABL-T315I to GNF-2 and Dasatinib. The PDLTCs were directly derived from BM cells of Ph + ALL patients cultured in a specific culture medium [9]. We recently established a novel PDLTC from a Ph + ALL patient harboring the BCR/ABL-T315I (KÖ) [8]. In this PDLTC, 50% of the cells harbor the BCR/ABL-T315I whereas the other 50% express unmutated BCR/ABL. We analyzed the response of increasing concentrations of PDLTCs from Ph + ALL patients expressing BCR/ABL-T315I (KÖ) to drug combinations. As negative controls, we used the PDLTCs from a Ph- ALL patient (HP). Cytotoxicity and proliferation were assessed at 72 h by XTT. At the dosages used, non-specific cytotoxic effects were not observed in the Ph- HP cells (Figure 3). Regarding the KÖ cells, the effects of GNF-2 and Dasatinib alone are attributable to the response of the 50% of the cell population, which express the unmutated BCR/ABL. The combination of GNF-2 and Dasatinib overcame the 50% effects of the single compounds and inhibited the proliferation of BCR/ABL-T315I-expressing PDLTCs with IC50 values of 1-1.25 μM and 100 nM (Figure 3).

In summary, these data show that the combination of allosteric inhibition and Dasatinib overcomes the resistance in primary PDLTCs from Ph + ALL patients harboring the BCR/ABL-T315I mutation.

### The combination of allosteric inhibition and dasatinib is able to abolish the transformation potential of BCR/ABL-T315I

**Figure 4:**
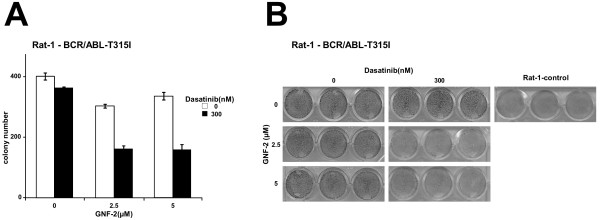
**Effects of GNF-2 and Dasatinib on Rat-1 cells expressing BCR/ABL-T315I.** (**A**) Transformation assays measuring colony formation in soft agar. Rat-1 cells were retrovirally transduced with the BCR/ABL-T315I construct and seeded at 10^4^ cells/well on soft agar in six-well plates in the presence of 2.5 or 5 μM GNF-2 and 300 nM Dasatinib. After 15 days, the colonies were counted. The means +/- SD of triplicates of two representative experiments are presented. (**B**) Focus formation assays. A total of 4x10^4^ infected Rat-1 cells were plated per well in 24-well plates, grown for 72 h to confluence and incubated for 12 additional days in the presence of 2.5 or 5 μM GNF-2 and 300 nM Dasatinib. The plates were then washed, dried and stained with crystal violet. One representative of each of the two experiments performed in triplicate is given (34X magnification).

We have shown recently that GNF-2 inhibits the transformation potential of unmutated BCR/ABL but not of BCR/ABL-T315I in untransformed fibroblasts [4]. Therefore, we asked the question of whether the combination of GNF-2 with Dasatinib is able to inhibit the transformation potential of BCR/ABL-T315I. The transformation potential of BCR/ABL-T315I in the presence of GNF-2 +/- Dasatinib was assessed using classical transformation assays for the detection of contact inhibition and anchorage-dependent growth in untransformed Rat-1 fibroblasts. Thus, we retro virally expressed BCR/ABL-T315I in Rat-1 cells. Empty-vector-transduced Rat-1 cells (mock) were used as controls. The transduction efficiency was assessed by the detection of GFP using flow cytometry. For each construct, triplicates of 10^3^ infected Rat-1 cells were placed on soft agar (anchorage-dependent growth) and in 6-well plates for the focus formation assay (contact inhibition). Colonies and “foci” stained with crystal violet were counted after 15 days. As shown in Figure 4A, only the combination of GNF-2 and Dasatinib was able to inhibit the colony formation and restore contact inhibition in Rat-1 cells expressing BCR/ABL-T315I (Figure 4A and B).

These data indicate that the combination of allosteric inhibitors with AKIs inhibits the transformation potential of BCR/ABL-T315I.

### GNF-2 cooperates with dasatinib to inhibit colony formation of hematopoietic stem and progenitors cells (HSPCs) harboring BCR/ABL-T315I in semi-solid medium

**Figure 5:**
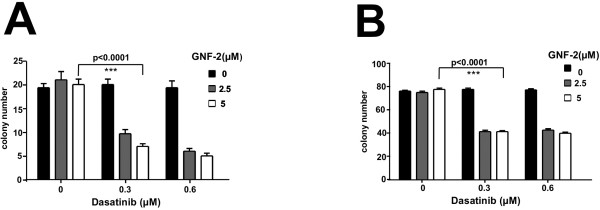
**Effects of GNF-2 and Dasatinib on HSPCs expressing BCR/ABL-T315I.** Sca1+ BM cells were retrovirally infected with BCR/ABL-T315. Infected cells were then plated in semi-solid medium in the presence of 2.5 or 5 μM GNF-2 and 0.3 or 0.6 μM Dasatinib (**A**) without cytokines and (**B**) with cytokines (mIL-3, mIL-6 and mSCF) to determine the effect of the drugs on HSPC colony formation in semi-solid medium. Colonies were counted after 10 days. The means +/- SD of triplicates from one representative experiment out of two performed are given.

To further confirm the synergistic effect of the combination of Dasatinib and GNF-2, we extended our investigation to a model of primary murine hematopoietic stem and progenitor cells expressing BCR/ABL. We studied the effects of the drug combination on the colony formation by BCR/ABL cells in semi-solid medium in the presence or absence of cytokines (mIL-3, mSCF and mIL-6). We transduced Sca1^+^ HSPCs with BCR/ABL-T315I, and plated the cells in methyl-cellulose with increasing concentrations of GNF-2 and Dasatinib. As shown in Figure 5A, colony formation was inhibited by Dasatinib and GNF-2 at concentrations of 300 nM and 2.5 μM, respectively, in the presence of cytokines. Interestingly, in the absence of cytokines, BCR/ABL-T315I formed compact colonies, which were inhibited efficiently with the combination of Dasatinib and GNF-2 at 300 nM and 2.5 μM, respectively (Figure 5B).

These data demonstrate that mHPSCs expressing the “gatekeeper” mutation T315I can be targeted efficiently by the combination of GNF-2 and Dasatinib.

## Discussion

The major therapeutic challenge in Ph + leukemia is to efficiently treat patients with BCR/ABL harboring the T315I mutation. The T315I mutation is the most resistant to inhibition because of a combination of several factors, including steric hindrance of drug binding, loss of a key hydrogen-bonding interaction with the T315 side-chain hydroxyl group exploited by Imatinib, Nilotinib and Dasatinib and potentially through increasing aberrant intrinsic kinase activity accompanied by aberrant substrate phosphorylation [13,14]. Unfortunately, T315I confers resistance not only against ABL kinase inhibitors but also against the allosteric inhibition by GNF-2 [11]. Allosteric inhibition is a novel approach for targeting BCR/ABL, which overcomes the resistance mediated by the T315I in combination with inhibition of oligomerization [4]. The fact that the competitive peptides for oligomerization inhibition are still far from clinical application led us to explore whether the allosteric inhibition could also improve the response of BCR/ABL-T315I to competitive ATP analogues.

GNF-2 and its analogues are non-ATP competitive ABL kinase inhibitors, which bind to the MBP in the kinase domain. It seems that the binding of GNF-2 to the MBP stabilizes the protein in an inhibited conformation resulting in a structural reorganization of ABL that disrupts the catalytic machinery located in the ATP-binding region [7]. Thus, one can speculate that GNF-2 introduces changes in the overall conformations of BCR/ABL-T315I, which renders the ATP-binding site more accessible to Dasatinib. This result is confirmed by recent biophysical studies showing that Dasatinib induces conformational changes in unmutated BCR/ABL but not in BCR/ABL-T315I. In contrast, GNF-5 leads to the same changes in both unmutated BCR/ABL and BCR/ABL-T315I [15].

An additive but not synergistic effect was shown for the combination of Nilotinib with GNF-2 or GNF-5 on BCR/ABL-T315I-related resistance. The stronger effects may be attributed to the fact that Dasatinib, originally developed as a SRC-kinase inhibitor, not only inhibits the BCR/ABL kinase but also targets a broader range of kinases compared to Nilotinib, the spectrum of which is mainly limited to ABL, c-KIT and PDGFR [16]. An additional effect of GNF-2 itself on SRC family kinases is unlikely. c-SRC is also myristoylated and harbors a putative MBP, which is involved in the regulation of c-SRC kinase activity, but in a manner very different from that for c-ABL [17].

Our data further establish allosteric inhibition as alternative or additional molecular therapy approach for the treatment of Ph^+^ leukemia. In fact, it not only overcomes the resistance mediated by the “gatekeeper” mutation T315I but also increases the response of unmutated BCR/ABL to AKI. In the clinical setting, this feature could contribute to a more efficient use of AKI at a lower dosage in “normally” responsive patients and the possibility to further increase dosage in patients early in the progression of disease, in the absence of BCR/ABL mutations, for whom dosage escalation is still a therapeutic option.

The results presented here contribute to the further development of allosteric inhibition for the molecular targeting of both unmutated BCR/ABL and BCR/ABL harboring the multi-resistance mutation T315I.

## Conclusions

Resistance and long-term tolerability of BCR/ABL inhibitors represent the major therapeutic challenge in Philadelphia Chromosome-positive (Ph+) leukemia. Advanced Ph + leukemia respond only transiently to ABL kinase inhibitors (AKI). Resistance is mostly caused by the acquisition of point mutations in BCR/ABL. The “gatekeeper” mutation T315I confers resistance against all available molecular therapy approaches. Conformational changes by allosteric inhibition increases the response of both unmutated BCR/ABL and BCR/ABL-T315I towards inhibition of oligomerization. Therefore we investigated whether the conformational changes induced by the allosteric inhibition also enhances the response towards the AKI Dasatinib in clinically relevant models of Ph + leukemia. Allosteric inhibition not only increased the response of unmutated BCR/ABL to Dasatinib but also contributed to overcome resistance of BCR/ABL-T315I in a synergistic manner in all models used.

Therefore allosteric inhibition may contribute to the optimization of the therapy of patients with both unmutated BCR/ABL or harboring resistance mutations such as the T315I.

## Competing interests

The authors declare that they have no competing interests.

## Author’s information

Afsar Ali Mian, Anna Metodieva both authors have to be considered first authors.

## Authors’ contribution

AAM carried out the studies on transduced Ba/F3 cells and participated in drafting the manuscript. AM performed the transformation assays on Rat-1 fibroblasts and on the murine HSCs. SB generated the PDLTCs harboring the BCR/ABL-T315I and carried out the experiments on these cells. MK and NR participated in the elaboration of the data. JM and YN participated in the design of the study and performed the statistical analysis. OGO participated in the design and coordination of the study. MR conceived of the study and wrote the paper. All authors read and approved the final manuscript.

## Pre-publication history

The pre-publication history for this paper can be accessed here:

http://www.biomedcentral.com/1471-2407/12/411/prepub
